# Associations Between Asthma Diagnosis/Asthma Exacerbation and Previous Proton-Pump Inhibitor use: A Nested Case-Control Study Using a National Health Screening Cohort

**DOI:** 10.3389/fphar.2022.888610

**Published:** 2022-06-30

**Authors:** Hyo Geun Choi, Chanyang Min, Dae Myoung Yoo, Bruce K. Tan, Joo-Hee Kim, Hwan Il Kim, Ji-Young Park, Sunghoon Park, Yong Il Hwang, Seung Hun Jang, Ki-Suck Jung

**Affiliations:** ^1^ Departments of Otorhinolaryngology-Head and Neck Surgery, Hallym University Sacred Heart Hospital, Hallym University College of Medicine, Anyang, South Korea; ^2^ Hallym Data Science Laboratory, Hallym University College of Medicine, Anyang, South Korea; ^3^ Department of Otolaryngology – Head and Neck Surgery, Northwestern University Feinberg School of Medicine, Chicago, IL, United States; ^4^ Division of Pulmonary, Allergy, and Critical Care Medicine, Department of Medicine, Hallym University Sacred Heart Hospital, Hallym University College of Medicine, Anyang, South Korea

**Keywords:** asthma, exacerbation (symptom flare up), cohort studies, proton pump inhibitor (PPI), inflammation

## Abstract

**Background:** Proton-pump inhibitors (PPIs) block acid secretion from gastric parietal cells; however, recent studies have reported that PPIs have antioxidant and anti-inflammatory properties in various cells. Newer PPIs are stronger inhibitors of acid secretion; however, the anti-inflammatory effects of these drugs have not been assessed. We evaluated anti-inflammatory effect of PPIs on the development of asthma/asthma exacerbation (AE) in a national health screening cohort.

**Methods:** This case-control study comprised 64,809 participants with asthma who were 1:1 matched with controls from the Korean National Health Insurance Service-Health Screening Cohort. Conditional logistic regression analysis was used to evaluate the effect of previous PPI use on an asthma diagnosis in all participants. Unconditional logistic regression was used to assess the effect of PPI use on AE in participants with asthma. These relationships were estimated in a subgroup analysis according to PPI generation.

**Results:** Overall, PPI use increased the risk of asthma diagnosis [adjusted odds ratio (aOR) = 1.29, 95% confidence interval (CI) = 1.23–1.35, *p* < 0.001]. Use of the first-generation PPIs was associated with asthma (aOR = 1.34, 95% CI = 1.18–1.52, *p* < 0.001), while use of second-generation PPIs was not (aOR = 0.97, 95% CI = 0.82–1.15, *p* = 0.748). In contrast, overall PPI use decreased the risk of AE in participants with asthma (aOR = 0.79, 95% CI = 0.75–0.84, *p* < 0.001), although this effect was observed only for second-generation PPIs (aOR = 0.76, 95% CI = 0.65–0.89, *p* = 0.001).

**Conclusion:** PPI use increased the risk for subsequent asthma diagnosis. However, this effect was confined to first-generation PPIs. Second-generation PPIs decreased the risk of AE.

## Introduction

Gastroesophageal reflux (GERD) and asthma, which are both common conditions, often coexist in the same patient. Estimates of the prevalence of GERD among patients with asthma have varied from 12.5% to 51.0% (Havemann et al., 2007; Kim et al., 2020a). A significant association between reflux disease and exacerbations in asthmatic patients has been observed [odds ratio (OR) = 4.9 95% Confidence Interval (CI) = 1.4–17.8] (Sontag et al., 2004; ten Brinke et al., 2005). Indeed, several randomized clinical trials (RCTs) have been performed to assess whether proton-pump inhibitors (PPIs) can improve asthma-related outcomes; however, there are some mixed findings (McDonnell et al., 2020). Patients with asthma who took PPIs for GERD showed improvement in peak expiratory flow rate and asthma-related quality of life scores but no significant benefit for asthma symptom scores and lung function (Littner et al., 2005; Kiljander et al., 2006; American Lung Association Asthma Clinical Research Centers et al., 2009). Therefore, current asthma guidelines recommend that anti-reflux therapy should be recommended to patients with asthma having concomitant reflux symptoms (Global Initiative for Asthma, 2020).

PPIs are considered the most effective therapy for relieving GERD symptoms. PPIs block gastric acid secretion by inhibiting H+, K+ ATPase of gastric parietal cells. Newer PPIs, such as esomeprazole, rabeprazole, and ilaprazole, known as second-generation PPIs, are more stable, and their plasma concentration is not strongly influenced by different cytochrome P450 enzyme activities; therefore, they are more potent at inhibiting acid secretion than first-generation PPIs (Kirchheiner et al., 2009; Yu et al., 2017).

In addition, PPIs have anti-inflammatory properties that might prevent inflammation in PPI-responsive eosinophilic esophagitis (EoE) (Kedika et al., 2009; Dellon et al., 2018). PPIs blocked the IL-4- and IL-13-stimulated increase in eotaxin mRNA and protein secretion through signal transducer and activator of transcription 6 (STAT6) in EoE (Zhang et al., 2012). In addition, PPIs reduce esophageal eosinophilia by restoring esophageal mucosal barrier integrity, which is important, as dilated intercellular spaces make the esophageal mucosa permeable to swallowed antigens linked to type 2 inflammation (Dellon et al., 2018). Considering that sensitization to ingested or inhaled allergens is necessary for the development of EoE (Furuta and Katzka, 2015), which is a common pathogenic feature of allergic asthma, PPI use could prevent asthma development or improve asthma-related outcomes. Previous studies demonstrated that PPIs reduced IL-13–induced eotaxin-3 expression via non-gastric H and K-ATPase and that newer PPIs more strongly inhibited eotaxin-3 secretion, suggesting that the anti-type 2 effect was well correlated with the potency of individual PPIs (Cortes et al., 2009; Min et al., 2017).

Based on these findings, we hypothesized that PPIs might be associated with a reduction in asthma diagnosis or asthma exacerbation (AE) in adulthood. To estimate the PPI effect on asthma diagnosis or asthma-related outcomes, we conducted a matched case-control study using a nationwide, population-based cohort in a Korean adult population. The primary objective was to estimate the association between PPI use and subsequent asthma diagnosis compared to that in control participants. We also analyzed the effect of PPIs on allergic asthma, as PPIs have anti-type 2 activities, and allergic asthma is a type 2-dominant inflammatory phenotype. The secondary objective was to evaluate the association between PPI use and AE compared to non-asthma exacerbation (NAE) among the participants with asthma. We further analyzed the effect of PPI generation on asthma diagnosis and AE to identify a relationship between acid-suppressive potency and the anti-inflammatory efficacy of PPIs.

## Methods

### Data Source and Ethical Consideration

We used the Korean National Health Insurance Service-Health Screening Cohort (NHIS-NSC) data for this study; a comprehensive description of this cohort is provided elsewhere (Kim et al., 2019; NHISS, 2020). Briefly, these data constitute approximately a million participants, 2.2% of the total eligible population, who were randomly sampled from the 2002 and followed for 11 years, until 2013. These data include information about participants’ demographics, health insurance claim codes, diagnostic codes, socio-economic status, and medical examination data. This study was approved by the Institutional Review Board (IRB) of Hallym University (IRB No: 2019-10-023), and the need for written informed consent was waived.

### Participant Selection

Among the 514,866 participants with 615,488,428 medical claim codes, participants were selected for the asthma group according to the definition in our study (n = 81,106). Asthma was defined if the participants from the cohort were diagnosed with asthma (J45, J46) based on the International Classification of Diseases, Tenth Revision (ICD-10). Among these individuals, we selected participants who were diagnosed with asthma more than two times by a physician and who were treated with asthma-related medications. Asthma medications include inhaled corticosteroids (ICS), ICS combined with long-acting β2-agonists (LABAs), short-acting β2-agonists (SABAs), systemic LABAs, leukotriene antagonists (LTRAs), xanthine derivatives, or systemic corticosteroids (Kim et al., 2013). Participants in the asthma group who were diagnosed with asthma in 2002 were removed to select asthma participants diagnosed for the first time (washout periods, n = 14,044). Other participants were selected for the control group (n = 433,760). In the control group, participants were excluded if they died before 2003 or had no records after 2003 (n = 34). In addition, the control participants were excluded if they were treated for J45 or J46 ICD-10 codes with no record of an asthma medication prescription (n = 108,730). Participants who were diagnosed with hiatal hernia, Zollinger-Ellison syndrome, systemic sclerosis, achalasia or pyloric obstruction, who underwent gastric surgery, and who had no record of total cholesterol/blood pressure/body mass index (BMI, kg/m^2^) were excluded. The asthma participants were 1:1 matched with control participants for age, sex, income, and region of residence. To minimize selection bias, the control participants were selected with random number generation. The index date of each asthma participant was set as the time of treatment of asthma. The index date for individual control participants was set as the index date of their matched asthma participant. Therefore, each matched asthma participant and control participant had the same index date. Using the above matching rules, 253,128 control participants were excluded and finally, 64,809 asthma participants were 1:1 matched with 64,809 control participants.

### Proton-Pump Inhibitor (Exposure)

PPI users were defined as participants who were prescribed PPIs within the previous year (365 days) before the index date. PPI users were classified into three categories as follows: 1) PPI prescription history, 2) the summation of PPI prescription dates, and 3) PPI prescription dates for each generation of PPIs.

Participants with a PPI prescription were grouped as current PPI users if they were prescribed PPIs within 30 days before the index date. If the participants were prescribed PPIs within 31–365 days before the index date, they were grouped as past PPI users. Others were grouped as PPI non-users.

The summation of PPI prescription dates was divided into four groups: 1) PPI non-users, 2) PPI prescription 1–29 days, 3) PPI prescription 30–89 days, and 4) PPI prescription ≥90 days in 1 year.

In the third category, the participants were recategorized into two categories according to the generations of PPIs (McNicholl et al., 2012). The first generation of PPIs consisted of pantoprazole, omeprazole, and lansoprazole. Second-generation PPIs were esomeprazole, dexlansoprazole, rabeprazole, and ilaprazole. PPI prescription dates were summed according to each generation of PPIs.

### Asthma (Outcome)

The asthma group included participants who were treated for asthma (ICD-10: J45) or status asthmaticus (J46) with asthma-related medications ≥2 times from 2002 through 2015. This definition was adapted from that in a previously validated study (Kim S. et al., 2013).

Among the asthma participants, AE was defined if asthma participants visited emergency medical doctors or had admissions that were not their first visit for asthma (Kim et al., 2021). Asthma participants who were prescribed steroids ≥20 mg a day ≥3 days within 2 weeks were also defined as having AE (Kim et al., 2021).

Allergic asthma was defined as having at least one of the following: 1) history of allergic rhinitis by ICD-10 code (J30) within 1 year or 2) therapy with oral antihistamines, leukotriene modifiers, intranasal corticosteroid spray, or intranasal antihistamines for ≥1 month within 1 year. All asthma patients who did not meet the definition of allergic asthma were classified as having non-allergic asthma (Robinson et al., 2021; Woo et al., 2021).

**FIGURE 1:**
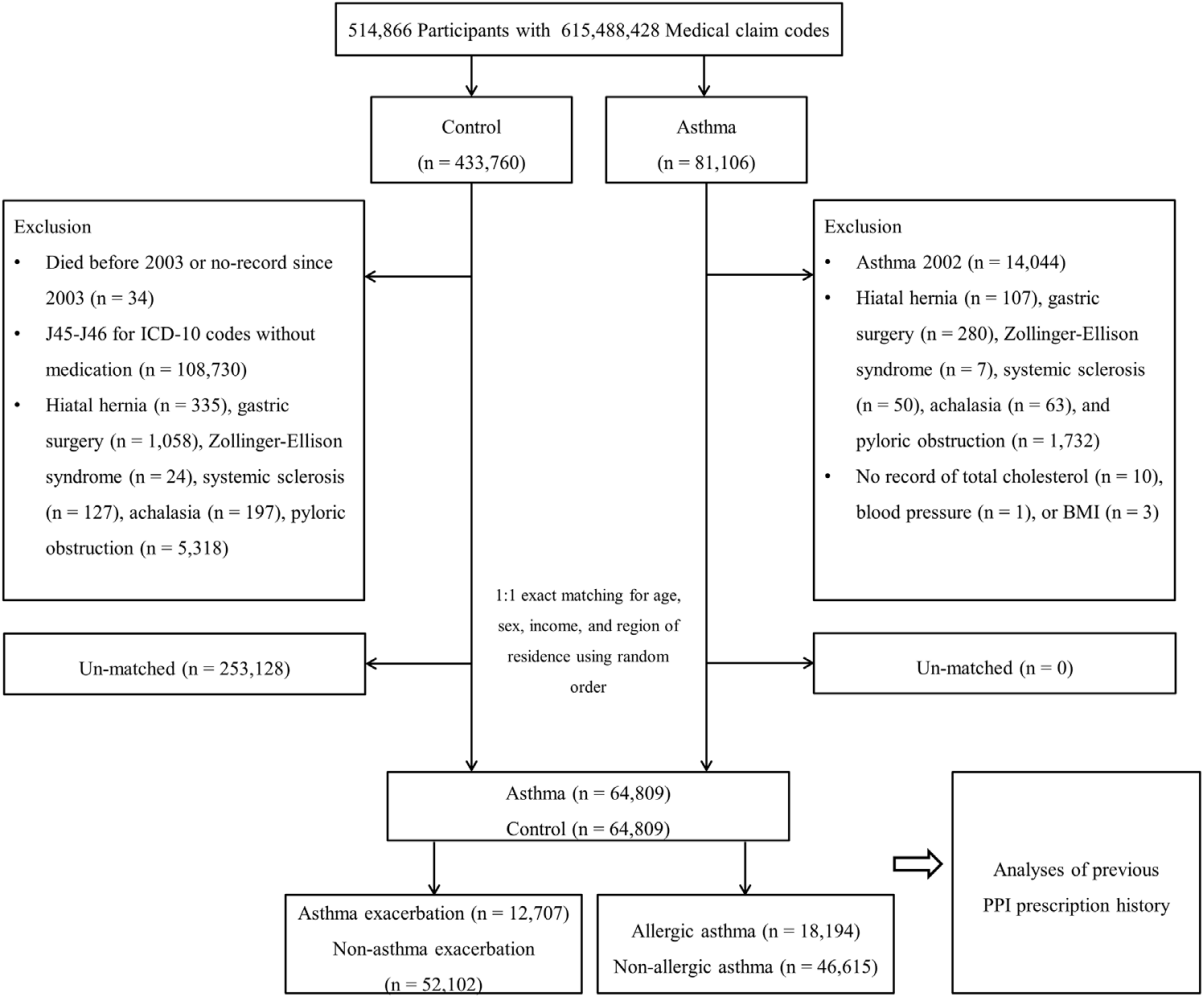
A schematic illustration of the participant selection process that was used in the present study. Of 514,866 participants, 64,809 asthma participants were matched with the same number of control participants for age, sex, income, and region of residence.

Based on the definition above, asthma participants were classified into AE (n = 12,707) and NAE (n = 52,102) groups and allergic asthma (n = 18,194) and non-allergic asthma (n = 46,615) groups. The history of previous PPI prescription was analyzed for an association with asthma by comparing asthma patients to the controls, the AE group to the NAE group, and the allergic asthma group to the non-allergic asthma group (Figure 1).

To evaluate the effects of PPI on AE, we set a new index date for AE and NAE. The new index date for AE was defined as the first exacerbation date. For NAE, a random date between the first asthma onset and the last day of follow-up was selected.

### Covariates

Age groups were divided into 5-years intervals, beginning at 40–44 years and ending at 85+ years (Kim et al., 2020a). Income groups were classified into five classes (class 1 [lowest income]-5 [highest income]). The area of residence was classified as urban/rural based on the definitions in a previous study (Kim et al., 2020a). Tobacco smoking, alcohol consumption, and obesity according to BMI were categorized the same way as in our previous study (Kim et al., 2020b).

Total cholesterol (mg/dl), systolic blood pressure (mmHg), diastolic blood pressure (mmHg), and fasting blood glucose (mg/dl) were measured. The Charlson Comorbidity Index (CCI) was applied excluding respiratory diseases. GERD and chronic obstructive pulmonary disease (COPD) were defined as in our previous study (Kim J. et al., 2013). Among GERD patients, GERD treatment within the 2 years (730 days) before the index date was quantified.

We summed each histamine-2 receptor antagonist (H2RA) and non-steroidal anti-inflammatory drug (NSAID) prescription date within a year (365 days) before the index date.

### Statistical Analyses

The demographic characteristics were compared between the asthma and control groups, and the differences were considered standardized difference (SDs).

To analyze the odds ratios (ORs) with 95% confidence intervals (CIs) of PPI prescription history for asthma, PPI prescription dates for asthma, and first generation/second generation PPI prescription dates for asthma, conditional logistic regression was used. In these analyses, crude and adjusted models were stratified for age, sex, income, and region of residence.

Regarding AE and allergic asthma, only asthma participants were included in the analyses. To analyze the odds ratios (ORs) with 95% confidence intervals (CIs) of PPI prescription history for AE/allergic asthma, PPI prescription dates for AE/allergic asthma, and first generation/second generation PPI prescription dates for AE/allergic asthma, unconditional logistic regression was used. In these analyses, various adjusted models were calculated.

Subgroup analyses were performed for the sensitivity analysis. In this study, we divided participants by age (<60 years old and ≥60 years old), sex (males and females), income (low income and high income), and region of residence (urban and rural).

SAS version 9.4 (SAS Institute Inc., Cary, NC, United States) was used for the statistical analysis. We performed two-tailed analyses, and a *p* value of <0.05 was considered statistically significant.

## Results

### Baseline Characteristics of the Study Participants

This study comprised 64,890 people with asthma and the same number of control participants. Because the patients were exactly matched for age group, sex, income level, and region of residence, their SD was 0. Other characteristics were not significantly different (SD ≤ 0.2, Table 1).

**TABLE 1:** General characteristics of participants.

Characteristics	Total participants		Standardized difference	Participants with asthma		Standardized difference
	Asthma	Control		AE	NAE	
Number	64,809	64,809		12,707	52,102	
Asthma exacerbation (n, %)	12,707 (19.6)	0 (0.0)	0.70	12,707 (100.0)	0 (0.0)	0.11
Allergic asthma (n, %)	18,194 (28.1)	0 (0.0)	0.88	6,722 (52.9)	11,472 (22.0)	0.67
Age (years, n, %)			0.00			0.22
40–44	2,119 (3.3)	2,119 (3.3)		206 (1.6)	226 (0.4)	
45–49	6,721 (10.4)	6,721 (10.4)		804 (6.3)	2,044 (3.9)	
50–54	9,804 (15.1)	9,804 (15.1)		1,431 (11.3)	6,844 (13.1)	
55–59	10,474 (16.2)	10,474 (16.2)		1,700 (13.4)	8,887 (17.1)	
60–64	10,602 (16.4)	10,602 (16.4)		1,870 (14.7)	8,586 (16.5)	
65–69	10,530 (16.3)	10,530 (16.3)		2,038 (16.0)	8,445 (16.2)	
70–74	8,035 (12.4)	8,035 (12.4)		1,958 (15.4)	8,052 (15.5)	
75–79	4,524 (7.0)	4,524 (7.0)		1,601 (12.6)	5,468 (10.5)	
80–84	1,683 (2.6)	1,683 (2.6)		807 (6.4)	2,729 (5.2)	
85+	317 (0.5)	317 (0.5)		292 (2.3)	821 (1.6)	
Sex (n, %)			0.00			0.00
Males	29,537 (45.6)	29,537 (45.6)		5,816 (45.8)	23,721 (45.5)	
Females	35,272 (54.4)	35,272 (54.4)		6,891 (54.2)	28,381 (54.5)	
Income (n, %)			0.00			0.07
1 (lowest)	11,551 (17.8)	11,551 (17.8)		2,451 (19.3)	9,099 (17.5)	
2	9,171 (14.2)	9,171 (14.2)		1,776 (14.0)	6,753 (13.0)	
3	10,293 (15.9)	10,293 (15.9)		2,002 (15.8)	7,915 (15.2)	
4	13,388 (20.7)	13,388 (20.7)		2,562 (20.2)	10,938 (21.0)	
5 (highest)	20,406 (31.5)	20,406 (31.5)		3,916 (30.8)	17,397 (33.4)	
Region of residence (n, %)			0.00			0.12
Urban	27,057 (41.8)	27,057 (41.8)		4,632 (36.5)	21,918 (42.1)	
Rural	37,752 (58.3)	37,752 (58.3)		8,075 (63.6)	30,184 (57.9)	
Total cholesterol level (mg/dl, mean, SD)	199.9 (38.7)	200.0 (38.5)	0.00	200.2 (39.4)	199.9 (38.5)	0.01
SBP (mmHg, mean, SD)	127.2 (17.3)	128.2 (17.9)	0.05	128.2 (18.0)	127.0 (17.1)	0.07
DBP (mmHg, mean, SD)	78.6 (10.9)	79.0 (11.2)	0.04	79.1 (11.2)	78.5 (10.8)	0.05
Fasting blood glucose level (mg/dl, mean, SD)	99.3 (28.6)	100.9 (33.0)	0.05	98.6 (29.4)	99.4 (28.4)	0.03
Obesity (n, %)^a^			0.11			0.09
Underweight	1,676 (2.6)	1,711 (2.6)		463 (3.6)	1,213 (2.3)	
Normal	21,216 (32.7)	23,881 (36.9)		4,136 (32.6)	17,080 (32.8)	
Overweight	17,097 (26.4)	17,554 (27.1)		3,177 (25.0)	13,920 (26.7)	
Obese I	22,300 (34.4)	19,831 (30.6)		4,367 (34.4)	17,933 (34.4)	
Obese II	2,520 (3.9)	1,832 (2.8)		564 (4.4)	1,956 (3.8)	
Smoking status (n, %)			0.03			0.11
Non-smoker	47,904 (73.9)	48,742 (75.2)		9,178 (72.2)	38,726 (74.3)	
Past smoker	6,307 (9.7)	5,986 (9.2)		1,067 (8.4)	5,240 (10.1)	
Current smoker	10,598 (16.4)	10,081 (15.6)		2,462 (19.4)	8,136 (15.6)	
Alcohol consumption (n, %)			0.03			0.08
<1 time a week	48,507 (74.9)	47,710 (73.6)		9,848 (77.5)	38,659 (74.2)	
≥1 time a week	16,302 (25.2)	17,099 (26.4)		2,859 (22.5)	13,443 (25.8)	
CCI score (score, n, %)			0.08			0.25
0	42,657 (65.8)	45,030 (69.5)		7,159 (56.3)	35,498 (68.1)	
1	9,594 (14.8)	8,235 (12.7)		2,252 (17.7)	7,342 (14.1)	
2	5,792 (8.9)	5,137 (7.9)		1,441 (11.3)	4,351 (8.4)	
≥3	6,766 (10.4)	6,407 (9.9)		1,855 (14.6)	4,911 (9.4)	
H2 blocker prescription dates (days, mean, SD)	34.7 (68.0)	22.2 (57.6)	0.20	50.9 (82.6)	42.6 (77.5)	0.10
NSAID prescription dates (days, mean, SD)	39.8 (66.4)	27.5 (58.5)	0.20	52.2 (76.6)	47.0 (73.0)	0.07
COPD (n, %)	16,166 (24.9)	2,143 (3.3)	0.65	5,467 (43.0)	10,699 (20.5)	0.50
GERD (n, %)	12,871 (19.9)	7,700 (11.9)	0.22	3,365 (26.5)	15,493 (29.7)	0.07
No. treated GERD patients (No., mean, SD)	0.8 (2.9)	0.5 (2.1)	0.14	1.3 (3.7)	1.5 (4.1)	0.05
PPI prescription history (n, %)			0.14			0.10
PPI non-user	55,964 (86.4)	58,851 (90.8)		9,854 (77.6)	38,817 (74.5)	
Past PPI user	6,745 (10.4)	4,730 (7.3)		1,990 (15.7)	10,190 (19.6)	
Current PPI user	2,100 (3.2)	1,228 (1.9)		863 (6.8)	3,095 (5.9)	
PPI prescription dates (days, mean, SD)	7.1 (31.5)	4.7 (25.6)	0.08	14.2 (47.8)	15.9 (50.4)	0.03
PPI prescription dates group (n, %)			0.14			0.07
PPI non-user	55,964 (86.4)	58,851 (90.8)		9,854 (77.6)	38,819 (74.5)	
1–29 days	4,787 (7.4)	3,180 (4.9)		1,405 (11.1)	6,731 (12.9)	
30–89 days	2,663 (4.1)	1,910 (3.0)		871 (6.9)	3,856 (7.4)	
≥90 days	1,395 (2.2)	868 (1.3)		577 (4.5)	2,696 (5.2)	
1st-generation PPI prescription dates (days, mean, SD)	4.5 (24.3)	3.0 (19.9)	0.07	8.5 (35.9)	8.4 (36.4)	0.00
1st-generation PPI prescription dates group (n, %)			0.11			0.02
PPI non-user	58,675 (90.5)	60,659 (93.6)		10,783 (84.9)	44,325 (85.1)	
1–29 days	3,413 (5.3)	2,264 (3.5)		998 (7.9)	4,169 (8.0)	
30–89 days	1,903 (2.9)	1,349 (2.1)		598 (4.7)	2,233 (4.3)	
≥90 days	818 (1.3)	537 (0.8)		328 (2.6)	1,375 (2.6)	
2nd-generation PPI prescription dates (days, mean, SD)	2.2 (17.4)	1.4 (14.7)	0.05	5.4 (29.6)	7.1 (32.8)	0.05
2nd-generation PPI prescription dates group (n, %)			0.09			0.12
PPI non-user	61,841 (95.4)	62,903 (97.1)		11,472 (90.3)	45,108 (86.6)	
1–29 days	1,705 (2.6)	1,052 (1.6)		695 (5.5)	3,907 (7.5)	
30–89 days	816 (1.3)	589 (0.9)		324 (2.6)	1,937 (3.7)	
≥90 days	447 (0.7)	265 (0.4)		216 (1.7)	1,150 (2.2)	

AE, asthma exacerbation; NAE, non-asthma exacerbation; CCI, Charlson Comorbidity Index; COPD, chronic obstructive pulmonary disease; DBP, diastolic blood pressure; GERD, gastroesophageal reflux disease; NSAID, non-steroidal anti-inflammatory drug; PPI, proton-pump inhibitor; SBP, systolic blood pressure; SD, standard deviation.

a BMI (body mass index, kg/m^2^) was categorized as <18.5 (underweight), ≥18.5 to <23 (normal), ≥23 to <25 (overweight), ≥25 to <30 (obese I), and ≥30 (obese II).

Among the 64,809 participants with asthma, 19.6% (n = 12,707) comprised the AE group, while the remaining patients (n = 52,102) comprised the NAE group. Their characteristics were not significantly different (SD ≤ 0.025) except for COPD (SD = 0.500).

### The Association Between PPI Use and Asthma Diagnosis

**FIGURE 2:**
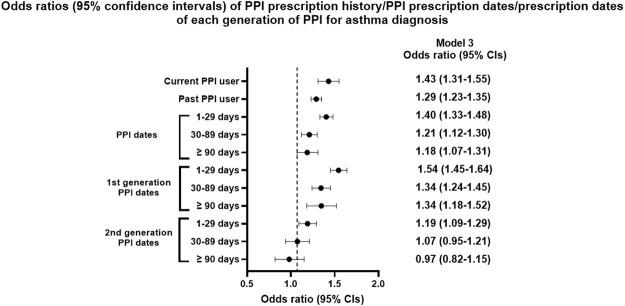
Odds ratios (95% confidence intervals) of PPI prescription history/PPI prescription dates/prescription dates of each generation of PPI for asthma diagnosis.

The numbers of current and past PPI users were higher among participants with asthma than among controls (3.2%/1.9% for current PPI users: 10.4%/7.3% for past PPI users, Table 1). The previous history of PPI prescription was related to asthma diagnosis in both current and past PPI users (*p* < 0.001, Table 2 and Figure 2). Regardless of the cumulative prescription days of PPIs, the OR for asthma was significantly increased in all models (*p* < 0.001).

**TABLE 2:** Odds ratios (95% confidence intervals) of PPI prescription history/PPI prescription dates/prescription dates of each generation of PPI for asthma.

Characteristics	Asthma (exposure/total, %)	Control (exposure/total, %)	Odds ratios (95% confidence intervals)							
			Crude^a^	*p*-value	Model 1^a^ ^,^ ^b^	*p*-value	Model 2^a^ ^,^ ^c^	*p*-value	Model 3^a^ ^,^ ^d^	*p*-value
PPI users										
Current PPI user	2,100/64,809 (3.2)	1,228/64,809 (1.9)	1.81 (1.68–1.94)	<0.001*	1.80 (1.67–1.93)	<0.001*	1.79 (1.67–1.92)	<0.001*	1.43 (1.31–1.55)	<0.001*
Past PPI user	6,745/64,809 (10.4)	4,730/64,809 (7.3)	1.50 (1.45–1.56)	<0.001*	1.50 (1.44–1.56)	<0.001*	1.50 (1.44–1.56)	<0.001*	1.29 (1.23–1.35)	<0.001*
PPI dates										
1–29 days	4,787/64,809 (7.4)	3,180/64,809 (4.9)	1.59 (1.52–1.66)	<0.001*	1.58 (1.51–1.65)	<0.001*	1.58 (1.51–1.66)	<0.001*	1.40 (1.33–1.48)	<0.001*
30–89 days	2,663/64,809 (4.1)	1,910/64,809 (3.0)	1.47 (1.39–1.56)	<0.001*	1.46 (1.38–1.55)	<0.001*	1.46 (1.37–1.55)	<0.001*	1.21 (1.12–1.30)	<0.001*
≥90 days	1,395/64,809 (2.2)	868/64,809 (1.3)	1.70 (1.56–1.85)	<0.001*	1.70 (1.56–1.85)	<0.001*	1.67 (1.53–1.82)	<0.001*	1.18 (1.07–1.31)	0.002*
PPI dates (1st generation)										
1–29 days	3,413/64,809 (5.3)	2,264/64,809 (3.5)	1.56 (1.48–1.65)	<0.001*	1.55 (1.47–1.64)	<0.001*	1.55 (1.47–1.64)	<0.001*	1.54 (1.45–1.64)	<0.001*
30–89 days	1,903/64,809 (2.9)	1,349/64,809 (2.1)	1.46 (1.36–1.57)	<0.001*	1.45 (1.36–1.56)	<0.001*	1.45 (1.35–1.55)	<0.001*	1.34 (1.24–1.45)	<0.001*
≥90 days	818/64,809 (1.3)	537/64,809 (0.8)	1.58 (1.42–1.76)	<0.001*	1.57 (1.41–1.76)	<0.001*	1.54 (1.38–1.72)	<0.001*	1.34 (1.18–1.52)	<0.001*
PPI dates (2nd generation)										
1–29 days	1,705/64,809 (2.6)	1,052/64,809 (1.6)	1.65 (1.53–1.79)	<0.001*	1.64 (1.52–1.77)	<0.001*	1.65 (1.53–1.79)	<0.001*	1.19 (1.09–1.29)	<0.001*
30–89 days	816/64,809 (1.3)	589/64,809 (0.9)	1.41 (1.27–1.57)	<0.001*	1.41 (1.26–1.56)	<0.001*	1.42 (1.27–1.58)	<0.001*	1.07 (0.95–1.21)	0.237
≥90 days	447/64,809 (0.7)	265/64,809 (0.4)	1.72 (1.48–2.01)	<0.001*	1.72 (1.48–2.00)	<0.001*	1.70 (1.46–1.98)	<0.001*	0.97 (0.82–1.15)	0.748

CCI, Charlson comorbidity index; COPD, chronic obstructive pulmonary disease; DBP, diastolic blood pressure; GERD, gastroesophageal reflux disease; NSAID, non-steroidal anti-inflammatory drug; PPI, proton-pump inhibitor; SBP, systolic blood pressure.

*Conditional logistic regression model, significance at *p* < 0.05.

a Models stratified by age, sex, income, and region of residence.

b Model 1 was adjusted for total cholesterol, SBP, DBP, and fasting blood glucose.

c Model 2 was adjusted for model 1 plus obesity, smoking, alcohol consumption, and CCI score.

d Model 3 was adjusted for model 2 plus NSAID dates, H2 blocker dates, COPD history, and number of treated GERD patients.

The OR for asthma with first generation of PPIs was significantly increased in all three models (*p* < 0.001). However, the OR for asthma with the second generation of PPIs was not increased in the fully adjusted model when cumulative prescription days were >30 days (OR = 1.07, 95% CI = 0.95–1.21, *p* = 0.237 for 30–89 days; OR = 0.97, 95% CI = 0.82–1.15, *p* = 0.748 for ≥90 days).

Subgroup analysis was performed according to obesity, smoking, alcohol consumption, and region of residence (Supplementary Tables S1, S2). Significant associations were noted between PPI users and asthma diagnosis for all subgroups (*p* < 0.001 for all).

When we confined PPI effects to allergic asthma, only current PPI users exhibited an association with asthma in all models (OR = 1.13, 95% CI = 1.02–1.25, *p* = 0.022, Table 3). First-generation PPI use ≥90 days was associated with an increased OR. However, no significant association was observed between second-generation PPIs and an allergic asthma diagnosis (OR = 0.97, 95% CI = 0.78–1.21, *p* = 0.769 for ≥90 days in model 7).

**TABLE 3:** Odds ratios (95% confidence intervals) of PPI prescription history/PPI prescription dates/prescription dates of each generation of PPI for allergic asthma in asthma patients.

Characteristics	Allergic asthma (exposure/total, %)	Non-allergic asthma (exposure/total, %)	Odds ratios (95% confidence intervals)							
			Model 4^a^	*p*-value	Model 5^b^	*p*-value	Model 6^c^	*p*-value	Model 7^d^	*p*-value
PPI users										
Current PPI user	643/18,194 (3.5)	1,457/46,615 (3.1)	1.11 (1.01–1.22)	0.037*	1.11 (1.01–1.22)	0.033*	1.12 (1.02–1.23)	0.019*	1.13 (1.02–1.25)	0.022*
Past PPI user	1,845/18,194 (10.1)	4,900/46,615 (10.5)	0.95 (0.90–1.01)	0.105	0.95 (0.90–1.01)	0.106	0.97 (0.91–1.02)	0.229	0.96 (0.91–1.02)	0.222
PPI dates										
1–29 days	1,291/18,194 (7.1)	3,496/46,615 (7.5)	0.94 (0.88–1.01)	0.078	0.94 (0.88–1.01)	0.074	0.95 (0.89–1.02)	0.155	0.96 (0.90–1.03)	0.279
30–89 days	762/18,194 (4.2)	1,901/46,615 (4.1)	1.01 (0.93–1.10)	0.854	1.01 (0.93–1.10)	0.823	1.02 (0.94–1.11)	0.630	1.02 (0.93–1.11)	0.739
≥90 days	435/18,194 (2.4)	960/46,615 (2.1)	1.12 (1.00–1.26)	0.051	1.13 (1.00–1.26)	0.044	1.14 (1.01–1.28)	0.030*	1.13 (0.99–1.28)	0.069
PPI dates (1st generation)										
1–29 days	959/18,194 (5.3)	2,454/46,615 (5.3)	1.00 (0.93–1.08)	0.990	1.00 (0.93–1.08)	0.988	1.00 (0.93–1.09)	0.917	1.00 (0.92–1.08)	0.916
30–89 days	569/18,194 (3.1)	1,334/46,615 (2.9)	1.08 (0.98–1.20)	0.122	1.08 (0.98–1.20)	0.118	1.09 (0.98–1.20)	0.106	1.07 (0.96–1.19)	0.215
≥90 days	272/18,194 (1.5)	546/46,615 (1.2)	1.24 (1.07–1.43)	0.005*	1.24 (1.07–1.44)	0.004*	1.25 (1.08–1.45)	0.003*	1.21 (1.04–1.42)	0.016*
PPI dates (2nd generation)										
1–29 days	435/18,194 (2.4)	1,270/46,615 (2.7)	0.87 (0.78–0.97)	0.011*	0.87 (0.78–0.97)	0.013*	0.89 (0.80–1.00)	0.048*	0.94 (0.84–1.06)	0.320
30–89 days	221/18,194 (1.2)	595/46,615 (1.3)	0.93 (0.79–1.08)	0.332	0.93 (0.80–1.09)	0.351	0.96 (0.82–1.12)	0.581	0.98 (0.83–1.15)	0.814
≥90 days	122/18,194 (0.7)	325/46,615 (0.7)	0.92 (0.75–1.14)	0.451	0.93 (0.75–1.14)	0.483	0.95 (0.77–1.17)	0.612	0.97 (0.78–1.21)	0.769

CCI, Charlson comorbidity index; COPD, chronic obstructive pulmonary disease; DBP, diastolic blood pressure; GERD, gastroesophageal reflux disease; NSAID, non-steroidal anti-inflammatory drug; PPI, proton-pump inhibitor; SBP, systolic blood pressure.

*Unconditional logistic regression model, significance at *p* < 0.05.

a Model 4 was adjusted for age, sex, income, and region of residence.

b Model 5 was adjusted for model 4 plus total cholesterol, SBP, DBP, and fasting blood glucose.

c Model 6 was adjusted for model 5 plus obesity, smoking, alcohol consumption, and CCI score.

d Model 7 was adjusted for model 6 plus NSAID dates, H2 blocker dates, COPD history, and number of treated GERD patients.

### The Association Between PPI Use and AE

**FIGURE 3:**
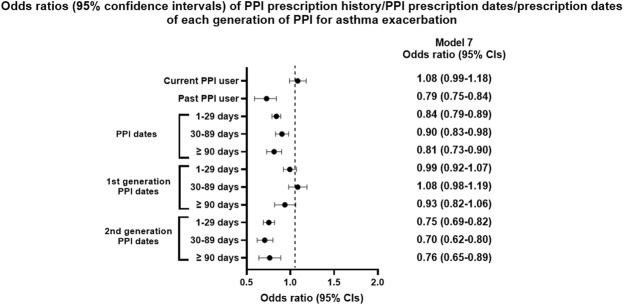
Odds ratios (95% confidence intervals) of PPI prescription history/PPI prescription dates/prescription dates of each generation of PPI for asthma exacerbation.

The proportion of current PPI users was higher in the AE group than in the NAE group (6.8%/5.9%), while past PPI users showed the opposite result (15.7%/19.6%, Table 1). Past PPI users were associated with a reduced OR for AE (*p* < 0.001), while current PPI users were not (Table 4 and Figure 3).

**TABLE 4:** Odds ratios (95% confidence intervals) of PPI prescription history/PPI prescription dates/prescription dates of each generation of PPI for asthma exacerbation.

Characteristics	Asthma exacerbation (exposure/total, %)	Non-asthma exacerbation (exposure/total, %)	Odds ratios (95% confidence intervals)							
			Model 4^a^	*p*-value	Model 5^b^	*p*-value	Model 6^c^	*p*-value	Model 7^d^	*p*-value
PPI users										
Current PPI user	863/12,707 (6.8)	3,095/52,102 (5.9)	1.07 (0.99–1.16)	0.076	1.08 (1.00–1.17)	0.052	1.06 (0.98–1.15)	0.133	1.08 (0.99–1.18)	0.086
Past PPI user	1,990/12,707 (15.7)	10,190/52,102 (19.6)	0.76 (0.72–0.80)	<0.001*	0.77 (0.73–0.81)	<0.001*	0.77 (0.73–0.81)	<0.001*	0.79 (0.75–0.84)	<0.001*
PPI dates										
1–29 days	1,405/12,707 (11.1)	6,731/52,102 (12.9)	0.82 (0.77–0.87)	<0.001*	0.82 (0.77–0.87)	<0.001*	0.83 (0.78–0.88)	<0.001*	0.84 (0.79–0.89)	<0.001*
30–89 days	871/12,707 (6.9)	3,856/52,102 (7.4)	0.88 (0.82–0.95)	0.001*	0.89 (0.82–0.96)	0.002*	0.89 (0.82–0.96)	0.002*	0.90 (0.83–0.98)	0.013*
≥90 days	577/12,707 (4.5)	2,696/52,102 (5.2)	0.82 (0.75–0.90)	<0.001*	0.82 (0.75–0.90)	<0.001*	0.80 (0.73–0.88)	<0.001*	0.81 (0.73–0.90)	<0.001*
PPI dates (1st generation)										
1–29 days	998/12,707 (7.9)	4,169/52,102 (8.0)	0.97 (0.91–1.05)	0.472	0.98 (0.91–1.05)	0.534	0.97 (0.90–1.05)	0.454	0.99 (0.92–1.07)	0.796
30–89 days	598/12,707 (4.7)	2,233/52,102 (4.3)	1.09 (0.99–1.20)	0.072	1.09 (1.00–1.20)	0.062	1.07 (0.97–1.17)	0.163	1.08 (0.98–1.19)	0.114
≥90 days	328/12,707 (2.6)	1,375/52,102 (2.6)	0.96 (0.85–1.08)	0.464	0.96 (0.85–1.08)	0.500	0.91 (0.81–1.03)	0.140	0.93 (0.82–1.06)	0.288
PPI dates (2nd generation)										
1–29 days	695/12,707 (5.5)	3,907/52,102 (7.5)	0.70 (0.64–0.76)	<0.001*	0.70 (0.64–0.76)	<0.001*	0.72 (0.67–0.79)	<0.001*	0.75 (0.69–0.82)	<0.001*
30–89 days	324/12,707 (2.6)	1,937/52,102 (3.7)	0.65 (0.58–0.74)	<0.001*	0.66 (0.58–0.74)	<0.001*	0.68 (0.60–0.76)	<0.001*	0.70 (0.62–0.80)	<0.001*
≥90 days	216/12,707 (1.7)	1,150/52,102 (2.2)	0.72 (0.62–0.83)	<0.001*	0.72 (0.62–0.84)	<0.001*	0.72 (0.62–0.83)	<0.001*	0.76 (0.65–0.89)	0.001*

CCI, Charlson comorbidity index; COPD, chronic obstructive pulmonary disease; DBP, diastolic blood pressure; GERD, gastroesophageal reflux disease; NSAID, non-steroidal anti-inflammatory drug; PPI, proton-pump inhibitor; SBP, systolic blood pressure.

*Unconditional logistic regression model, significance at *p* < 0.05.

a Model 4 was adjusted for age, sex, income, and region of residence.

b Model five was adjusted for model 4 plus total cholesterol, SBP, DBP, and fasting blood glucose.

c Model 6 was adjusted for model five plus obesity, smoking, alcohol consumption, and CCI score.

d Model 7 was adjusted for model 6 plus NSAID dates, H2 blocker dates, COPD history, and number of treated GERD patients.

Cumulative prescription days of PPIs were also associated with reduced AE in all models (*p* < 0.05). The PPI effect on AE was observed only with the second generation PPIs in all models (*p* < 0.001), while it was not with the first generation PPIs.

Subgroup analyses showed that PPI use was associated with reduced AE in all subgroups except underweight (*p* < 0.001 for all except underweight, Supplementary Table S4). However, the GERD subgroup showed an interaction effect between PPI use and asthma AE (*p* = 0.043). In all subgroups except underweight, second-generation PPI users had a decreased OR for AE.

## Discussion

This study found that previous and current PPI use was associated with incident asthma in a Korean adult population. However, PPI use decreased AE defined by oral steroid bursts, ER visits, or hospitalizations, among the participants with asthma. Subgroup analyses showed that the association between PPIs and asthma/AE was different according to PPI generation. Only first-generation PPIs were associated with an increased risk for asthma diagnosis, and second-generation PPIs were associated with a decreased risk for AE among participants with asthma.

Previous association studies were primarily performed in children whose mothers used PPIs and/or H2RAs during pregnancy, causing their offspring to develop asthma. Meta-analyses have revealed an increased risk of asthma development in childhood with the use of any acid-suppressive medications (relative risk = 1.36, 95% CI = 1.16–1.65), H2RAs (hazard ratio [HR] = 1.46, 95% CI = 1.29–1.65), or PPIs (HR = 1.30, 95% CI = 1.07–1.56) (Devine et al., 2017). A recent Swedish nationwide cohort study showed a significantly increased risk of asthma among children who started PPI treatment than among those who did not start PPIs (HR = 1.57, 95% CI = 1.49–1.64) (Wang et al., 2021). In the adult population, only one study investigated the association between asthma incidence and PPI use ≥90 days, and the overall incidence of asthma was greater in the PPI cohort than in the non-PPI cohort (HR = 1.76, 95% CI = 1.64–1.88) (Wang et al., 2020). Our study found that PPI use increased the risk for subsequent asthma diagnosis, with an OR of 1.43; however, this association was observed with only first-generation PPIs.

One postulated mechanism linking PPI use to asthma development is that PPIs could cause imbalance in symbiotic and pathological gut species. For infants or children, a greater abundance of a specific bacterium or reduced bacterial diversity was associated with the development of allergic diseases (Zimmermann et al., 2019). A prospective study in children showed that PPI treatment induced dysbiosis and bacterial overgrowth, suggesting that PPI use could cause dysbiosis in the gut. This change may promote type 2 inflammation in the immune system (Belei et al., 2018). An adult population-based cohort study demonstrated a lower abundance of gut commensals and lower microbial diversity in PPI users than in PPI non-users (Jackson et al., 2016; Le Bastard et al., 2018; Naito et al., 2018). Gut microbiota could induce immune responses in distal organs, especially the respiratory tract (Barcik et al., 2020). Recent studies have reported that significant relationship between gut microbiota composition and asthma phenotype/endotype in adults (Begley et al., 2018; Zou et al., 2021). A direct link between PPI use and gut microbiome/asthma has not been reported yet in adulthood; however, we postulate that PPI use might be associated with alterations to gut microbiota, which affect the development of asthma. Further epidemiologic and mechanistic evidence is essential to support the link between the PPI effect on the gut microbiome and subsequent asthma development, considering different PPIs, doses, and durations in childhood and adulthood.

In this study, previous PPI use decreased the risk of AE in participants with asthma, which was greater in participants who took PPIs for ≥90 days (OR = 0.81, 95% CI = 0.73–0.90). A potential mechanism for the protective effect of PPIs against AE might be their anti-type 2 or anti-inflammatory action. PPIs significantly inhibited IL-13-induced eotaxin-3 expression in nasal epithelium, and patients with chronic rhinosinusitis (CRS) taking PPIs showed lower *in vivo* eotaxin-3 levels than those not taking PPIs (Min et al., 2017). In addition, PPIs favourably control the oxidant–antioxidant system by scavenging reactive oxygen species in airways (Yoshida et al., 2000; Sasaki et al., 2005; Ghebremariam et al., 2015). Recent transcriptomic data demonstrated that PPIs exert pharmacological actions in regulating immune, vascular endothelial, and airway epithelial biology; they do not just hinder the final-step of IL-13 induced inflammatory responses (Kedika et al., 2009; Ghebre and Raghu, 2016). Therefore, we postulated that the decreased OR for AE in PPI users could be due to the pleiotropic and anti-type 2 properties of PPIs.

This study found that PPI effects on asthma or AE differed according to the generation of the drug. Second-generation PPIs significantly decreased the diagnosis of allergic asthma with an OR of 0.87–0.89 and were associated with a decrease in AE (OR = 0.76, 95% CI = 0.65–0.89, ≥90 days), indicating that PPIs, especially second generation, might be beneficial for allergic asthma or AE, which has a type 2 inflammatory milieu. Pharmacokinetic studies showed that second-generation PPIs were more effective in suppressing gastric acid secretion because they rapidly converted to the active metabolite sulfenamide and had better reactivity with cysteines in H+, K+ ATPase on gastric parietal cells (Besancon et al., 1997; Yu et al., 2017). An adult population study showed that rabeprazole did not increase the risk of asthma (aHR = 1.04, 95% CI = 0.94–1.15) compared to first-generation PPIs (aHR = 1.38, 95% CI = 1.26–1.50 for omeprazole; aHR = 1.15, 95% CI = 1.04–1.24 for pantoprazole) (Wang et al., 2020). The latest transcriptome data in EoE revealed that substantial overlap was observed in gene expression with omeprazole and esomeprazole treatment (Rochman et al., 2021). However, esomeprazole induced more significant changes in gene expression than omeprazole, suggesting that individual PPIs may have different effects on inflammation and homeostasis in airways. These mechanistic and epidemiological data could partly explain our main results.

This study has some limitations. First, this study used health insurance data; therefore, we applied an operational definition of asthma/AE, which is not as accurate as the definitions in RCTs and registry-based studies. Second, we used prescription days, but actual drug exposure could not be monitored in this study. Third, we adjusted for several variables related to PPI use to minimize confounding effects between PPIs and asthma/AE; however, as it had a retrospective-design, unmeasured confounding effects could not be excluded. Fourth, our data differed from an already published RCT, in which no additional benefit was seen for PPI use to control asthma symptoms in asymptomatic GERD and asthma patients (American Lung Association Asthma Clinical Research Centers et al., 2009). However, the current study demonstrated that regardless of GERD status, PPI use decreased AE in participants with asthma.

This study has the following strengths. First, we included a large population of participants treated with PPIs, with ample statistical power in the primary analysis. Second, we adjusted for variables related to PPI use or asthma, such as NSAID or H2RA prescriptions, obesity, GERD, and COPD. Third, we analyzed PPI use and AE in participants with asthma, which has not been done in other studies. We found that second-generation PPIs were associated with a decreased risk of AE, in accord with recent research data on anti-inflammatory effects and PPIs.

In conclusion, we found that PPI use was associated with an increased risk of asthma. However, this effect was confined to first-generation PPIs. Second-generation PPIs decreased the risk of AE in participants with asthma.

## Data Availability

The data analyzed in this study is subject to the following licenses/restrictions: The datasets generated and analyzed during the current study are not publicly available due to the need for approval from the Korean National Health Insurance Service Committee, but they are available from the corresponding author on reasonable request. Requests to access these datasets should be directed to J-HK, luxjhee@gmail.com.

## Abbreviations

AE, asthma exacerbation; EoE, eosinophilic esophagitis; GERD, gastroesophageal reflux disease; H2RA, histamine-2 receptor antagonist; HR, hazard ratios; NAE, non-asthma exacerbation; ORs, odds ratios; PPIs, proton pump inhibitors; RCTs, randomized clinical trials.
