# Supplementary material for: Associations Between Asthma Diagnosis/Asthma Exacerbation and Previous Proton-Pump Inhibitor use: A Nested Case-Control Study Using a National Health Screening Cohort
Source: Front Pharmacol. 2022 Jun 30;13:888610. doi: 10.3389/fphar.2022.888610 (PMC9279665; doi:10.3389/fphar.2022.888610)
Supplement: Supplementary file 1 [file DataSheet1.docx]

**S1 Table** Subgroup analyses regarding odds ratio (95% confidence intervals) of PPI prescription history/PPI prescription dates/each generation of PPI prescription history for asthma according to age, sex, income, and region of residence

| Characteristics | | Asthma | Control | ORs (95% CIs) | | P for  interaction |
| --- | --- | --- | --- | --- | --- | --- |
|  |  | (exposure/total, %) | (exposure/total, %) | Model 3†‡ | P-value |  |
| User of PPI | |  |  |  |  |  |
|  | Age < 60 years old (n = 58,236) | 3,302/29,118 (11.3) | 2,144/29,118 (7.4) | 1.28 (1.20-1.37) | <0.001* | 0.013* |
|  | Age ≥ 60 years old (n = 71,382) | 5,543/35,691 (15.5) | 3,814/35,691 (10.7) | 1.32 (1.25-1.39) | <0.001* |  |
|  | Males (n = 59,074) | 4,217/29,537 (14.3) | 2,762/29,537 (9.4) | 1.33 (1.25-1.42) | <0.001* | 0.086 |
|  | Females (n = 70,544) | 4,628/35,272 (13.1) | 3,196/35,272 (9.1) | 1.29 (1.21-1.38) | <0.001* |  |
|  | Low income (n = 62,030) | 4,211/31,015 (13.6) | 2,826/31,015 (9.1) | 1.30 (1.23-1.38) | <0.001* | 0.516 |
|  | High income (n = 67,588) | 4,634/33,794 (13.7) | 3,132/33,794 (9.3) | 1.32 (1.25-1.40) | <0.001* |  |
|  | Urban (n = 54,114) | 3,489/27,057 (12.9) | 2,355/27,057 (8.7) | 1.29 (1.20-1.37) | <0.001* | 0.838 |
|  | Rural (n = 75,504) | 5,356/37,752 (14.2) | 3,603/37,752 (9.5) | 1.41 (1.33-1.48) | <0.001* |  |
| PPI dates (per 1 year) | |  |  |  |  |  |
|  | Age < 60 years old (n = 58,236) | N/A | N/A | 1.39 (0.93-2.07) | 0.109 | <0.001* |
|  | Age ≥ 60 years old (n = 71,382) | N/A | N/A | 1.37 (1.12-1.68) | 0.002* |  |
|  | Males (n = 59,074) | N/A | N/A | 1.53 (1.19-1.96) | 0.001* | 0.029* |
|  | Females (n = 70,544) | N/A | N/A | 1.21 (0.95-1.52) | 0.117 |  |
|  | Low income (n = 62,030) | N/A | N/A | 1.28 (1.01-1.62) | 0.045* | 0.319 |
|  | High income (n = 67,588) | N/A | N/A | 1.70 (1.32-2.18) | <0.001* |  |
|  | Urban (n = 54,114) | N/A | N/A | 1.40 (1.04-1.89) | 0.025* | 0.219 |
|  | Rural (n = 75,504) | N/A | N/A | 0.71 (0.58-0.87) | 0.001* |  |
| User of PPI (1st generation) | |  |  |  |  |  |
|  | Age < 60 years old (n = 58,236) | 2,320/29,118 (8.0) | 1,538/29,118 (5.3) | 1.18 (1.09-1.27) | <0.001* | 0.019* |
|  | Age ≥ 60 years old (n = 71,382) | 3,814/35,691 (10.7) | 2,612/35,691 (7.3) | 1.32 (1.24-1.41) | <0.001* |  |
|  | Males (n = 59,074) | 2,968/29,537 (10.1) | 1,946/29,537 (6.6) | 1.25 (1.16-1.34) | <0.001* | 0.132 |
|  | Females (n = 70,544) | 3,166/35,272 (9.0) | 2,204/35,272 (6.3) | 1.47 (1.37-1.57) | <0.001* |  |
|  | Low income (n = 62,030) | 2,981/31,015 (9.6) | 1,966/31,015 (6.3) | 1.24 (1.16-1.32) | <0.001* | 0.557 |
|  | High income (n = 67,588) | 3,153/33,794 (9.3) | 2,184/33,794 (6.5) | 1.31 (1.23-1.40) | <0.001* |  |
|  | Urban (n = 54,114) | 2,380/27,057 (8.8) | 1,661/27,057 (6.1) | 1.36 (1.27-1.47) | <0.001* | 0.276 |
|  | Rural (n = 75,504) | 3,754/37,752 (9.9) | 2,489/37,752 (6.6) | 1.27 (1.19-1.35) | <0.001* |  |
| User of PPI (2nd generation) | |  |  |  |  |  |
|  | Age < 60 years old (n = 58,236) | 985/29,118 (3.4) | 593/29,118 (2.0) | 1.34 (1.19-1.50) | <0.001* | 0.030* |
|  | Age ≥ 60 years old (n = 71,382) | 1,983/35,691 (5.6) | 1,313/35,691 (3.7) | 1.70 (1.56-1.85) | <0.001* |  |
|  | Males (n = 59,074) | 1,395/29,537 (4.7) | 884/29,537 (3.0) | 1.36 (1.23-1.51) | <0.001* | 0.604 |
|  | Females (n = 70,544) | 1,573/35,272 (4.5) | 1,022/35,272 (2.9) | 2.32 (2.09-2.57) | <0.001* |  |
|  | Low income (n = 62,030) | 1,380/31,015 (4.5) | 882/31,015 (2.8) | 1.40 (1.27-1.54) | <0.001* | 0.300 |
|  | High income (n = 67,588) | 1,588/33,794 (4.7) | 1,024/33,794 (3.0) | 1.62 (1.48-1.78) | <0.001* |  |
|  | Urban (n = 54,114) | 1,162/27,057 (4.3) | 718/27,057 (2.7) | 1.48 (1.33-1.65) | <0.001* | 0.242 |
|  | Rural (n = 75,504) | 1,806/37,752 (4.8) | 1,188/37,752 (3.2) | 1.33 (1.21-1.46) | <0.001* |  |

Abbreviations: CI, confidence interval; CCI, Charlson comorbidity index; COPD, chronic obstructive pulmonary disease; DBP, diastolic blood pressure; GERD, gastro-esophageal reflux disease; NSAID, non-steroidal anti-inflammatory drug; OR, odds ratio; PPI, proton pump inhibitor; SBP, systolic blood pressure

* Conditional logistic regression model, Significance at P < 0.05

† Models stratified by age, sex, income, and region of residence.

‡ Model 3 was adjusted for total cholesterol, SBP, DBP, fasting blood glucose, obesity, smoking, alcohol consumption, CCI score, NSAID dates, H2 blocker dates, COPD history, and number of GERD treated

**S2 Table** Subgroup analyses regarding odds ratio (95% confidence intervals) of PPI prescription history/PPI prescription dates/each generation of PPI prescription history for asthma according to obesity, smoking state, alcohol consumption state, COPD history, and GERD history

| Characteristics | | Asthma | Control | ORs (95% CIs) | | P for  interaction |
| --- | --- | --- | --- | --- | --- | --- |
|  |  | (exposure/total, %) | (exposure/total, %) | Model 7‡ | P-value |  |
| User of PPI | |  |  |  |  |  |
|  | Underweight (n = 3,387) | 193/1,676 (11.5) | 169/1,711 (9.9) | 1.10 (0.83-1.44) | 0.509 | 0.876 |
|  | Normal weight (n = 45,097) | 2,877/21,216 (13.6) | 2,105/23,881 (8.8) | 1.38 (1.29-1.48) | <0.001* |  |
|  | Overweight (n = 34,651) | 2,330/17,097 (13.6) | 1,617/17,554 (9.2) | 1.35 (1.25-1.46) | <0.001* |  |
|  | Obese (n = 46,483) | 3,445/24,820 (13.9) | 2,067/21,663 (9.5) | 1.33 (1.24-1.42) | <0.001* |  |
|  | Non-smoker (n = 96,646) | 6,383/47,904 (13.3) | 4,438/48,742 (9.1) | 1.33 (1.27-1.40) | <0.001* | 0.027* |
|  | Past and current smoker (n = 32,972) | 2,462/16,905 (14.6) | 1,520/16,067 (9.5) | 1.40 (1.29-1.51) | <0.001* |  |
|  | Alcohol consumption > 1 time a week (n = 96,217) | 6,208/48,507 (12.8) | 4,076/47,710 (8.5) | 1.35 (1.28-1.41) | <0.001* | 0.920 |
|  | Alcohol consumption ≤ 1 time a week (n = 33,401) | 2,637/16,302 (16.2) | 1,882/17,099 (11.0) | 1.33 (1.24-1.43) | <0.001* |  |
|  | Non-COPD (n = 111,309) | 6,793/48,643 (14.0) | 5,698/62,666 (9.1) | 1.37 (1.31-1.43) | <0.001* | <0.001* |
|  | COPD (n = 18,309) | 2,052/16,166 (12.7) | 260/2,143 (12.1) | 1.03 (0.89-1.19) | 0.714 |  |
|  | Non-GERD (n = 109,047) | 2,984/51,938 (5.8) | 2,477/57,109 (4.3) | 1.29 (1.22-1.37) | <0.001* | <0.001* |
|  | GERD (n = 20,571) | 5,861/12,871 (45.5) | 3,481/7,700 (45.2) | 1.04 (0.98-1.11) | 0.211 |  |
| PPI dates (per 1 year) | |  |  |  |  |  |
|  | Underweight (n = 3,387) | N/A | N/A | 0.88 (0.29-2.71) | 0.826 | 0.839 |
|  | Normal weight (n = 45,097) | N/A | N/A | 1.38 (1.01-1.89) | 0.040* |  |
|  | Overweight (n = 34,651) | N/A | N/A | 1.63 (1.16-2.30) | 0.005* |  |
|  | Obese (n = 46,483) | N/A | N/A | 1.23 (0.95-1.60) | 0.123 |  |
|  | Non-smoker (n = 96,646) | N/A | N/A | 1.31 (1.07-1.60) | 0.008 | 0.020* |
|  | Past and current smoker (n = 32,972) | N/A | N/A | 1.46 (1.04-2.04) | 0.027 |  |
|  | Alcohol consumption > 1 time a week (n = 96,217) | N/A | N/A | 1.20 (0.99-1.47) | 0.069 | 0.095 |
|  | Alcohol consumption ≤ 1 time a week (n = 33,401) | N/A | N/A | 1.77 (1.27-2.46) | 0.001 |  |
|  | Non-COPD (n = 111,309) | N/A | N/A | 1.35 (1.13-1.61) | 0.001 | 0.001* |
|  | COPD (n = 18,309) | N/A | N/A | 1.20 (0.67-2.16) | 0.547 |  |
|  | Non-GERD (n = 109,047) | N/A | N/A | 1.84 (1.36-2.49) | <0.001* | <0.001* |
|  | GERD (n = 20,571) | N/A | N/A | 0.90 (0.74-1.10) | 0.309 |  |
| User of PPI (1st generation) | |  |  |  |  |  |
|  | Underweight (n = 3,387) | 138/1,676 (8.2) | 133/1,711 (7.8) | 0.87 (0.63-1.18) | 0.360 | 0.393 |
|  | Normal weight (n = 45,097) | 2,002/21,216 (9.4) | 1,464/23,881 (6.1) | 1.28 (1.18-1.38) | <0.001* |  |
|  | Overweight (n = 34,651) | 1,597/17,097 (9.3) | 1,134/17,554 (6.5) | 1.23 (1.12-1.34) | <0.001* |  |
|  | Obese (n = 46,483) | 2,397/24,820 (9.7) | 1,419/21,663 (6.6) | 1.27 (1.18-1.37) | <0.001* |  |
|  | Non-smoker (n = 96,646) | 4,421/47,904 (9.2) | 3,097/48,742 (6.4) | 1.24 (1.18-1.31) | <0.001* | 0.052 |
|  | Past and current smoker (n = 32,972) | 1,713/16,905 (10.1) | 1,053/16,067 (6.6) | 1.29 (1.18-1.42) | <0.001* |  |
|  | Alcohol consumption > 1 time a week (n = 96,217) | 4,434/48,507 (9.1) | 2,916/47,710 (6.1) | 1.26 (1.20-1.33) | <0.001* | 0.576 |
|  | Alcohol consumption ≤ 1 time a week (n = 33,401) | 1,700/16,302 (10.4) | 1,234/17,099 (7.2) | 1.21 (1.11-1.32) | <0.001* |  |
|  | Non-COPD (n = 111,309) | 4,613/48,643 (9.5) | 3,965/62,666 (6.3) | 1.26 (1.21-1.33) | <0.001* | <0.001* |
|  | COPD (n = 18,309) | 1,521/16,166 (9.4) | 185/2,143 (8.6) | 1.07 (0.90-1.26) | 0.451 |  |
|  | Non-GERD (n = 109,047) | 2,009/51,938 (3.9) | 1,691/57,109 (3.0) | 1.22 (1.14-1.31) | <0.001* | <0.001* |
|  | GERD (n = 20,571) | 4,125/12,871 (32.1) | 2,459/7,700 (31.9) | 0.99 (0.93-1.05) | 0.731 |  |
| User of PPI (2nd generation) | |  |  |  |  |  |
|  | Underweight (n = 3,387) | 63/1,676 (3.8) | 41/1,711 (2.4) | 1.99 (1.25-3.16) | 0.004* | 0.218 |
|  | Normal weight (n = 45,097) | 951/21,216 (4.5) | 665/23,881 (2.8) | 1.39 (1.24-1.56) | <0.001* |  |
|  | Overweight (n = 34,651) | 800/17,097 (4.7) | 515/17,554 (2.9) | 1.41 (1.25-1.60) | <0.001* |  |
|  | Obese (n = 46,483) | 1,154/24,820 (4.6) | 685/21,663 (3.2) | 1.30 (1.17-1.44) | <0.001* |  |
|  | Non-smoker (n = 96,646) | 2,087/47,904 (4.4) | 1,373/48,742 (2.8) | 1.37 (1.27-1.48) | <0.001* | 0.582 |
|  | Past and current smoker (n = 32,972) | 881/16,905 (5.2) | 533/16,067 (3.3) | 1.39 (1.22-1.57) | <0.001* |  |
|  | Alcohol consumption > 1 time a week (n = 96,217) | 1,892/48,507 (3.9) | 1,170/47,710 (2.5) | 1.37 (1.26-1.48) | <0.001* | 0.893 |
|  | Alcohol consumption ≤ 1 time a week (n = 33,401) | 1,076/16,302 (6.6) | 736/17,099 (4.3) | 1.36 (1.22-1.51) | <0.001* |  |
|  | Non-COPD (n = 111,309) | 2,412/48,643 (5.0) | 1,813/62,666 (2.9) | 1.41 (1.32-1.51) | <0.001* | <0.001* |
|  | COPD (n = 18,309) | 556/16,166 (3.4) | 93/2,143 (4.3) | 0.76 (0.60-0.96) | 0.023 |  |
|  | Non-GERD (n = 109,047) | 685/51,938 (1.3) | 591/57,109 (1.0) | 1.34 (1.20-1.51) | <0.001* | <0.001* |
|  | GERD (n = 20,571) | 2,283/12,871 (17.7) | 1,315/7,700 (17.1) | 1.13 (1.04-1.22) | 0.003 |  |

Abbreviations: CI, confidence interval; CCI, Charlson comorbidity index; COPD, chronic obstructive pulmonary disease; DBP, diastolic blood pressure; GERD, gastro-esophageal reflux disease; NSAID, non-steroidal anti-inflammatory drug; OR, odds ratio; PPI, proton pump inhibitor; SBP, systolic blood pressure

* Un-conditional logistic regression model, Significance at P < 0.05

† Model 7 was adjusted for age, sex, income, region, total cholesterol, SBP, DBP, fasting blood glucose, obesity, smoking, alcohol consumption, CCI score, NSAID dates, H2 blocker dates, and COPD history, and number of GERD treated

**S3 Table** Subgroup analyses regarding odds ratio (95% confidence intervals) of PPI prescription history/PPI prescription dates/each generation of PPI prescription history for allergic asthma in asthma patients according to age, sex, income, region of residence, obesity, smoking state, alcohol consumption state, COPD history, and GERD history

| Characteristics | | Allergic asthma | Non-allergic asthma | ORs (95% CI) | | P for  interaction |
| --- | --- | --- | --- | --- | --- | --- |
|  |  | (exposure/total, %) | (exposure/total, %) | Model 7‡ | P-value |  |
| User of PPI | |  |  |  |  |  |
|  | Age < 60 years old (n = 29,118) | 920/7,789 (11.8) | 2,382/21,329 (11.2) | 1.02 (0.94-1.12) | 0.605 | 0.134 |
|  | Age ≥ 60 years old (n = 35,691) | 1,568/10,405 (15.1) | 3,975/25,286 (15.7) | 0.98 (0.91-1.05) | 0.470 |  |
|  | Males (n = 29,537) | 1,303/8,953 (14.6) | 2,914/20,584 (14.2) | 1.04 (0.96-1.13) | 0.307 | 0.214 |
|  | Females (n = 35,272) | 1,185/9,241 (12.8) | 3,443/26,031 (13.2) | 0.96 (0.89-1.04) | 0.315 |  |
|  | Low income (n = 31,015) | 1,210/8,866 (13.7) | 3,001/22,149 (13.6) | 1.01 (0.94-1.10) | 0.728 | 0.319 |
|  | High income (n = 33,794) | 1,278/9,328 (13.7) | 3,356/24,466 (13.7) | 0.98 (0.91-1.06) | 0.633 |  |
|  | Urban (n = 27,057) | 990/7,428 (13.3) | 2,499/19,629 (12.7) | 1.04 (0.95-1.13) | 0.410 | 0.111 |
|  | Rural (n = 37,752) | 1,498/10,766 (13.9) | 3,858/26,986 (14.3) | 0.97 (0.91-1.04) | 0.436 |  |
|  | Underweight (n = 1,676) | 57/556 (10.3) | 136/1,120 (12.1) | 0.89 (0.62-1.29) | 0.552 | 0.887 |
|  | Normal weight (n = 21,216) | 806/5,999 (13.4) | 2,071/15,217 (13.6) | 1.00 (0.90-1.10) | 0.935 |  |
|  | Overweight (n = 17,097) | 678/4,808 (14.1) | 1,652/12,289 (13.4) | 1.05 (0.95-1.17) | 0.343 |  |
|  | Obese (n = 24,820) | 947/6,831 (13.9) | 2,498/17,989 (13.9) | 0.97 (0.89-1.06) | 0.556 |  |
|  | Non-smoker (n = 47,904) | 1,730/12,987 (13.3) | 4,653/34,917 (13.3) | 0.99 (0.93-1.06) | 0.727 | 0.887 |
|  | Past and current smoker (n = 16,905) | 758/5,207 (14.6) | 1,704/11,698 (14.6) | 1.03 (0.93-1.14) | 0.614 |  |
|  | Alcohol consumption > 1 time a week (n = 48,507) | 1,775/13,720 (12.9) | 4,433/34,787 (12.7) | 1.00 (0.94-1.07) | 0.992 | 0.549 |
|  | Alcohol consumption ≤ 1 time a week (n = 16,302) | 713/4,474 (15.9) | 1,924/11,828 (16.3) | 1.00 (0.90-1.11) | 0.948 |  |
|  | Non-COPD (n = 48,643) | 1,672/11,255 (14.9) | 5,121/37,388 (13.7) | 1.06 (0.99-1.13) | 0.097 | <0.001* |
|  | COPD (n = 16,166) | 816/6,939 (11.8) | 1,236/9,227 (13.4) | 0.86 (0.78-0.95) | 0.004* |  |
|  | Non-GERD (n = 51,938) | 810/14,437 (5.6) | 2,174/37,501 (5.8) | 0.97 (0.89-1.05) | 0.449 | 0.962 |
|  | GERD (n = 12,871) | 1,678/3,757 (44.7) | 4,183/9,114 (45.9) | 0.99 (0.91-1.07) | 0.744 |  |
| PPI dates (per 1 year) | |  |  |  |  |  |
|  | Age < 60 years old (n = 29,118) | N/A | N/A | 1.12 (0.70-1.81) | 0.634 | 0.278 |
|  | Age ≥ 60 years old (n = 35,691) | N/A | N/A | 1.07 (0.83-1.37) | 0.607 |  |
|  | Males (n = 29,537) | N/A | N/A | 1.12 (0.83-1.52) | 0.466 | 0.793 |
|  | Females (n = 35,272) | N/A | N/A | 1.04 (0.75-1.43) | 0.817 |  |
|  | Low income (n = 31,015) | N/A | N/A | 1.10 (0.81-1.49) | 0.557 | 0.886 |
|  | High income (n = 33,794) | N/A | N/A | 1.06 (0.78-1.46) | 0.704 |  |
|  | Urban (n = 27,057) | N/A | N/A | 1.10 (0.77-1.58) | 0.594 | 0.495 |
|  | Rural (n = 37,752) | N/A | N/A | 1.07 (0.81-1.40) | 0.655 |  |
|  | Underweight (n = 1,676) | N/A | N/A | 1.47 (0.35-6.21) | 0.603 | 0.935 |
|  | Normal weight (n = 21,216) | N/A | N/A | 1.00 (0.66-1.50) | 0.983 |  |
|  | Overweight (n = 17,097) | N/A | N/A | 1.52 (0.99-2.33) | 0.058 |  |
|  | Obese (n = 24,820) | N/A | N/A | 0.93 (0.67-1.31) | 0.683 |  |
|  | Non-smoker (n = 47,904) | N/A | N/A | 1.00 (0.77-1.30) | 0.988 | 0.504 |
|  | Past and current smoker (n = 16,905) | N/A | N/A | 1.32 (0.89-1.97) | 0.169 |  |
|  | Alcohol consumption > 1 time a week (n = 48,507) | N/A | N/A | 1.08 (0.83-1.39) | 0.573 | 0.736 |
|  | Alcohol consumption ≤ 1 time a week (n = 16,302) | N/A | N/A | 1.13 (0.74-1.72) | 0.580 |  |
|  | Non-COPD (n = 48,643) | N/A | N/A | 1.18 (0.91-1.53) | 0.220 | 0.013* |
|  | COPD (n = 16,166) | N/A | N/A | 0.87 (0.59-1.29) | 0.495 |  |
|  | Non-GERD (n = 51,938) | N/A | N/A | 1.00 (0.65-1.54) | 0.994 | 0.404 |
|  | GERD (n = 12,871) | N/A | N/A | 1.10 (0.85-1.43) | 0.479 |  |
| User of PPI (1st generation) | |  |  |  |  |  |
|  | Age < 60 years old (n = 29,118) | 677/7,789 (8.7) | 1,643/21,329 (7.7) | 1.07 (0.97-1.19) | 0.170 | 0.081 |
|  | Age ≥ 60 years old (n = 35,691) | 1,123/10,405 (10.8) | 2,691/25,286 (10.6) | 1.01 (0.94-1.10) | 0.739 |  |
|  | Males (n = 29,537) | 943/8,953 (10.5) | 2,025/20,584 (9.8) | 1.05 (0.96-1.15) | 0.271 | 0.891 |
|  | Females (n = 35,272) | 857/9,241 (9.3) | 2,309/26,031 (8.9) | 1.04 (0.95-1.13) | 0.446 |  |
|  | Low income (n = 31,015) | 897/8,866 (10.1) | 2,084/22,149 (9.4) | 1.06 (0.97-1.16) | 0.202 | 0.604 |
|  | High income (n = 33,794) | 903/9,328 (9.7) | 2,250/24,466 (9.2) | 1.03 (0.94-1.12) | 0.582 |  |
|  | Urban (n = 27,057) | 713/7,428 (9.6) | 1,667/19,629 (8.5) | 1.11 (1.00-1.22) | 0.045* | 0.055 |
|  | Rural (n = 37,752) | 1,087/10,766 (10.1) | 2,667/26,986 (9.9) | 1.00 (0.93-1.09) | 0.958 |  |
|  | Underweight (n = 1,676) | 40/556 (7.2) | 98/1,120 (8.8) | 0.84 (0.56-1.28) | 0.421 | 0.765 |
|  | Normal weight (n = 21,216) | 597/5,999 (10.0) | 1,405/15,217 (9.2) | 1.08 (0.96-1.20) | 0.194 |  |
|  | Overweight (n = 17,097) | 489/4,808 (10.2) | 1,108/12,289 (9.0) | 1.11 (0.99-1.26) | 0.081 |  |
|  | Obese (n = 24,820) | 674/6,831 (9.9) | 1,723/17,989 (9.6) | 0.99 (0.89-1.09) | 0.817 |  |
|  | Non-smoker (n = 47,904) | 1,259/12,987 (9.7) | 3,162/34,917 (9.1) | 1.05 (0.97-1.13) | 0.232 | 0.396 |
|  | Past and current smoker (n = 16,905) | 541/5,207 (10.4) | 1,172/11,698 (10.0) | 1.03 (0.92-1.16) | 0.585 |  |
|  | Alcohol consumption > 1 time a week (n = 48,507) | 1,342/13,720 (9.8) | 3,092/34,787 (8.9) | 1.08 (1.00-1.16) | 0.044* | 0.039* |
|  | Alcohol consumption ≤ 1 time a week (n = 16,302) | 458/4,474 (10.2) | 1,242/11,828 (10.5) | 0.95 (0.84-1.08) | 0.430 |  |
|  | Non-COPD (n = 48,643) | 1,176/11,255 (10.5) | 3,437/37,388 (9.2) | 1.10 (1.02-1.18) | 0.014* | <0.001* |
|  | COPD (n = 16,166) | 624/6,939 (9.0) | 897/9,227 (9.7) | 0.92 (0.82-1.03) | 0.133 |  |
|  | Non-GERD (n = 51,938) | 580/14,437 (4.0) | 1,429/37,501 (3.8) | 1.03 (0.93-1.14) | 0.553 | 0.340 |
|  | GERD (n = 12,871) | 1,220/3,757 (32.5) | 2,905/9,114 (31.9) | 1.02 (0.94-1.11) | 0.620 |  |
| User of PPI (2nd generation) | |  |  |  |  |  |
|  | Age < 60 years old (n = 29,118) | 258/7,789 (3.3) | 727/21,329 (3.4) | 0.98 (0.84-1.15) | 0.820 | 0.487 |
|  | Age ≥ 60 years old (n = 35,691) | 520/10,405 (5.0) | 1,463/25,286 (5.8) | 0.95 (0.85-1.05) | 0.309 |  |
|  | Males (n = 29,537) | 398/8,953 (4.5) | 997/20,584 (4.8) | 0.98 (0.86-1.11) | 0.749 | 0.746 |
|  | Females (n = 35,272) | 380/9,241 (4.1) | 1,193/26,031 (4.6) | 0.95 (0.83-1.07) | 0.378 |  |
|  | Low income (n = 31,015) | 368/8,866 (4.2) | 1,012/22,149 (4.6) | 0.97 (0.85-1.11) | 0.660 | 0.304 |
|  | High income (n = 33,794) | 410/9,328 (4.4) | 1,178/24,466 (4.8) | 0.95 (0.84-1.07) | 0.366 |  |
|  | Urban (n = 27,057) | 305/7,428 (4.1) | 857/19,629 (4.4) | 0.98 (0.85-1.13) | 0.759 | 0.467 |
|  | Rural (n = 37,752) | 473/10,766 (4.4) | 1,333/26,986 (4.9) | 0.94 (0.84-1.06) | 0.327 |  |
|  | Underweight (n = 1,676) | 20/556 (3.6) | 43/1,120 (3.8) | 1.20 (0.65-2.20) | 0.564 | 0.589 |
|  | Normal weight (n = 21,216) | 249/5,999 (4.2) | 702/15,217 (4.6) | 0.96 (0.82-1.13) | 0.650 |  |
|  | Overweight (n = 17,097) | 221/4,808 (4.6) | 579/12,289 (4.7) | 1.02 (0.86-1.21) | 0.856 |  |
|  | Obese (n = 24,820) | 288/6,831 (4.2) | 866/17,989 (4.8) | 0.90 (0.78-1.04) | 0.165 |  |
|  | Non-smoker (n = 47,904) | 520/12,987 (4.0) | 1,567/34,917 (4.5) | 0.94 (0.84-1.04) | 0.242 | 0.884 |
|  | Past and current smoker (n = 16,905) | 258/5,207 (5.0) | 623/11,698 (5.3) | 1.02 (0.86-1.20) | 0.845 |  |
|  | Alcohol consumption > 1 time a week (n = 48,507) | 494/13,720 (3.6) | 1,398/34,787 (4.0) | 0.92 (0.82-1.03) | 0.134 | 0.357 |
|  | Alcohol consumption ≤ 1 time a week (n = 16,302) | 284/4,474 (6.4) | 792/11,828 (6.7) | 1.05 (0.90-1.22) | 0.527 |  |
|  | Non-COPD (n = 48,643) | 563/11,255 (5.0) | 1,849/37,388 (5.0) | 0.98 (0.88-1.09) | 0.686 | 0.038* |
|  | COPD (n = 16,166) | 215/6,939 (3.1) | 341/9,227 (3.7) | 0.86 (0.72-1.04) | 0.114 |  |
|  | Non-GERD (n = 51,938) | 151/14,437 (1.1) | 534/37,501 (1.4) | 0.79 (0.66-0.96) | 0.014* | 0.626 |
|  | GERD (n = 12,871) | 627/3,757 (16.7) | 1,656/9,114 (18.2) | 1.00 (0.90-1.11) | 0.978 |  |

Abbreviations: CI, confidence interval; CCI, Charlson comorbidity index; COPD, chronic obstructive pulmonary disease; DBP, diastolic blood pressure; GERD, gastro-esophageal reflux disease; NSAID, non-steroidal anti-inflammatory drug; OR, odds ratio; PPI, proton pump inhibitor; SBP, systolic blood pressure

* Un-conditional logistic regression model, Significance at P < 0.05

† Model 7 was adjusted for age, sex, income, region, total cholesterol, SBP, DBP, fasting blood glucose, obesity, smoking, alcohol consumption, CCI score, NSAID dates, H2 blocker dates, and COPD history, and number of GERD treated

S4 Table Subgroup analyses regarding odds ratio (95% confidence intervals) of PPI prescription history/PPI prescription dates/each generation of PPI prescription history for asthma exacerbation in asthma patients according to age, sex, income, region of residence, obesity, smoking state, alcohol consumption state, COPD history, and GERD history

| Characteristics | | Asthma exacerbation | Non-asthma exacerbation | ORs (95% CI) | | P-value  for  interaction |
| --- | --- | --- | --- | --- | --- | --- |
|  |  | (exposure/total, %) | (exposure/total, %) | Model 7‡ | P-value |  |
| User of PPI | |  |  |  |  | <0.001* |
|  | Age < 60 years old (n = 22,142) | 726/4,141 (17.5) | 3,922/18,001 (21.8) | 0.84 (0.76-0.92) | <0.001* |  |
|  | Age ≥ 60 years old (n = 42,667) | 2,127/8,566 (24.8) | 9,363/34,101 (27.5) | 0.88 (0.83-0.94) | <0.001* |  |
|  | Males (n = 29,537) | 1,310/5,816 (22.5) | 5,841/23,721 (24.6) | 0.88 (0.82-0.95) | 0.002* | 0.186 |
|  | Females (n = 35,272) | 1,543/6,891 (22.4) | 7,444/28,381 (26.2) | 0.82 (0.77-0.88) | <0.001* |  |
|  | Low income (n = 29,996) | 1,384/6,229 (22.2) | 6,067/23,767 (25.5) | 0.85 (0.79-0.91) | <0.001* | 0.958 |
|  | High income (n = 34,813) | 1,469/6,478 (22.7) | 7,218/28,335 (25.5) | 0.86 (0.80-0.92) | <0.001* |  |
|  | Urban (n = 26,550) | 964/4,632 (20.8) | 5,367/21,918 (24.5) | 0.81 (0.75-0.89) | <0.001* | 0.479 |
|  | Rural (n = 38,259) | 1,889/8,075 (23.4) | 7,918/30,184 (26.2) | 0.87 (0.82-0.93) | <0.001* |  |
|  | Underweight (n = 1,676) | 86/463 (18.6) | 290/1,213 (23.9) | 0.76 (0.56-1.03) | 0.081 | 0.256 |
|  | Normal weight (n = 21,216) | 908/4,136 (22.0) | 4,264/17,080 (25.0) | 0.85 (0.77-0.93) | <0.001* |  |
|  | Overweight (n = 17,097) | 713/3,177 (22.4) | 3,643/13,920 (26.2) | 0.83 (0.75-0.92) | <0.001* |  |
|  | Obese (n = 24,820) | 1,146/4,931 (23.2) | 5,088/19,889 (25.6) | 0.88 (0.81-0.95) | 0.002* |  |
|  | Non-smoker (n = 47,904) | 2,092/9,178 (22.8) | 9,890/38,726 (25.5) | 0.86 (0.81-0.91) | <0.001* | 0.188 |
|  | Past and current smoker (n = 16,905) | 761/3,529 (21.6) | 3,395/13,376 (25.4) | 0.83 (0.75-0.91) | <0.001* |  |
|  | Alcohol consumption > 1 time a week (n = 48,507) | 2,217/9,848 (22.5) | 9,804/38,659 (25.4) | 0.86 (0.81-0.91) | <0.001* | 0.423 |
|  | Alcohol consumption ≤ 1 time a week (n = 16,302) | 636/2,859 (22.3) | 3,481/13,443 (25.9) | 0.83 (0.74-0.92) | <0.001* |  |
|  | Non-COPD (n = 48,643) | 1,621/7,240 (22.4) | 10,399/41,403 (25.1) | 0.87 (0.81-0.92) | <0.001* | 0.065 |
|  | COPD (n = 16,166) | 1,232/5,467 (22.5) | 2,886/10,699 (27.0) | 0.82 (0.76-0.90) | <0.001* |  |
|  | Non-GERD (n = 45,951) | 979/9,342 (10.5) | 4,068/36,609 (11.1) | 0.90 (0.83-0.97) | 0.008* | 0.043* |
|  | GERD (n = 18,858) | 1,874/3,365 (55.7) | 9,217/15,493 (59.5) | 0.86 (0.80-0.93) | <0.001* |  |
| PPI dates (per 1 year) | |  |  |  |  |  |
|  | Age < 60 years old (n = 22,142) | NA | NA | 0.79 (0.52-1.22) | 0.292 | <0.001* |
|  | Age ≥ 60 years old (n = 42,667) | NA | NA | 0.76 (0.64-0.92) | 0.004* |  |
|  | Males (n = 29,537) | NA | NA | 0.89 (0.70-1.13) | 0.341 | 0.044* |
|  | Females (n = 35,272) | NA | NA | 0.63 (0.50-0.80) | <0.001* |  |
|  | Low income (n = 29,996) | NA | NA | 0.73 (0.57-0.93) | 0.011* | 0.643 |
|  | High income (n = 34,813) | NA | NA | 0.77 (0.61-0.98) | 0.032* |  |
|  | Urban (n = 26,550) | NA | NA | 0.60 (0.44-0.81) | 0.001* | 0.257 |
|  | Rural (n = 38,259) | NA | NA | 0.84 (0.68-1.02) | 0.084 |  |
|  | Underweight (n = 1,676) | NA | NA | 0.39 (0.14-1.11) | 0.077 | 0.244 |
|  | Normal weight (n = 21,216) | NA | NA | 0.76 (0.55-1.04) | 0.081 |  |
|  | Overweight (n = 17,097) | NA | NA | 0.73 (0.51-1.03) | 0.075 |  |
|  | Obese (n = 24,820) | NA | NA | 0.82 (0.63-1.05) | 0.110 |  |
|  | Non-smoker (n = 47,904) | NA | NA | 0.72 (0.59-0.88) | 0.002* | 0.868 |
|  | Past and current smoker (n = 16,905) | NA | NA | 0.82 (0.60-1.11) | 0.199 |  |
|  | Alcohol consumption > 1 time a week (n = 48,507) | NA | NA | 0.75 (0.62-0.91) | 0.004* | 0.932 |
|  | Alcohol consumption ≤ 1 time a week (n = 16,302) | NA | NA | 0.74 (0.52-1.06) | 0.101 |  |
|  | Non-COPD (n = 48,643) | NA | NA | 0.67 (0.53-0.84) | 0.001* | 0.591 |
|  | COPD (n = 16,166) | NA | NA | 0.88 (0.68-1.12) | 0.293 |  |
|  | Non-GERD (n = 45,951) | NA | NA | 0.87 (0.65-1.16) | 0.349 | 0.031* |
|  | GERD (n = 18,858) | NA | NA | 0.75 (0.61-0.92) | 0.006* |  |
| User of PPI (1st generation) | |  |  |  |  |  |
|  | Age < 60 years old (n = 22,142) | 497/4,141 (12.0) | 2,276/18,001 (12.6) | 1.01 (0.90-1.13) | 0.935 | 0.123 |
|  | Age ≥ 60 years old (n = 42,667) | 1,427/8,566 (16.7) | 5,501/34,101 (16.1) | 1.02 (0.95-1.10) | 0.530 |  |
|  | Males (n = 29,537) | 908/5,816 (15.6) | 3,438/23,721 (14.5) | 1.05 (0.96-1.14) | 0.327 | 0.166 |
|  | Females (n = 35,272) | 1,016/6,891 (14.7) | 4,339/28,381 (15.3) | 0.97 (0.90-1.05) | 0.467 |  |
|  | Low income (n = 29,996) | 953/6,229 (15.3) | 3,548/23,767 (14.9) | 1.03 (0.95-1.12) | 0.467 | 0.243 |
|  | High income (n = 34,813) | 971/6,478 (15.0) | 4,229/28,335 (14.9) | 0.98 (0.91-1.07) | 0.685 |  |
|  | Urban (n = 26,550) | 647/4,632 (14.0) | 3,058/21,918 (14.0) | 1.00 (0.91-1.11) | 0.942 | 0.773 |
|  | Rural (n = 38,259) | 1,277/8,075 (15.8) | 4,719/30,184 (15.6) | 1.01 (0.94-1.09) | 0.831 |  |
|  | Underweight (n = 1,676) | 60/463 (13.0) | 169/1,213 (13.9) | 0.93 (0.65-1.33) | 0.681 | 0.825 |
|  | Normal weight (n = 21,216) | 634/4,136 (15.3) | 2,484/17,080 (14.5) | 1.06 (0.96-1.18) | 0.251 |  |
|  | Overweight (n = 17,097) | 470/3,177 (14.8) | 2,132/13,920 (15.3) | 0.95 (0.85-1.07) | 0.421 |  |
|  | Obese (n = 24,820) | 760/4,931 (15.4) | 2,992/19,889 (15.0) | 1.01 (0.92-1.11) | 0.833 |  |
|  | Non-smoker (n = 47,904) | 1,390/9,178 (15.1) | 5,794/38,726 (15.0) | 1.00 (0.93-1.07) | 0.994 | 0.995 |
|  | Past and current smoker (n = 16,905) | 534/3,529 (15.1) | 1,983/13,376 (14.8) | 1.03 (0.92-1.15) | 0.640 |  |
|  | Alcohol consumption > 1 time a week (n = 48,507) | 1,492/9,848 (15.2) | 5,794/38,659 (15.0) | 1.01 (0.94-1.08) | 0.818 | 0.922 |
|  | Alcohol consumption ≤ 1 time a week (n = 16,302) | 432/2,859 (15.1) | 1,983/13,443 (14.8) | 1.00 (0.88-1.13) | 0.982 |  |
|  | Non-COPD (n = 48,643) | 1,060/7,240 (14.6) | 5,958/41,403 (14.4) | 1.03 (0.96-1.11) | 0.442 | 0.075 |
|  | COPD (n = 16,166) | 864/5,467 (15.8) | 1,819/10,699 (17.0) | 0.97 (0.88-1.06) | 0.477 |  |
|  | Non-GERD (n = 45,951) | 651/9,342 (7.0) | 2,275/36,609 (6.2) | 1.04 (0.95-1.15) | 0.371 | 0.004* |
|  | GERD (n = 18,858) | 1,273/3,365 (37.8) | 5,502/15,493 (35.5) | 1.06 (0.98-1.15) | 0.151 |  |
| User of PPI (2nd generation) | |  |  |  |  |  |
|  | Age < 60 years old (n = 22,142) | 297/4,141 (7.2) | 1,967/18,001 (10.9) | 0.77 (0.67-0.88) | <0.001* | <0.001* |
|  | Age ≥ 60 years old (n = 42,667) | 938/8,566 (11.0) | 5,027/34,101 (14.7) | 0.76 (0.70-0.82) | <0.001* |  |
|  | Males (n = 29,537) | 544/5,816 (9.4) | 3,019/23,721 (12.7) | 0.77 (0.70-0.86) | <0.001* | 0.348 |
|  | Females (n = 35,272) | 691/6,891 (10.0) | 3,975/28,381 (14.0) | 0.72 (0.66-0.79) | <0.001* |  |
|  | Low income (n = 29,996) | 585/6,229 (9.4) | 3,184/23,767 (13.4) | 0.72 (0.65-0.79) | <0.001* | 0.573 |
|  | High income (n = 34,813) | 650/6,478 (10.0) | 3,810/28,335 (13.5) | 0.76 (0.69-0.84) | <0.001* |  |
|  | Urban (n = 26,550) | 412/4,632 (8.9) | 2,884/21,918 (13.2) | 0.67 (0.60-0.76) | <0.001* | 0.125 |
|  | Rural (n = 38,259) | 823/8,075 (10.2) | 4,110/30,184 (13.6) | 0.78 (0.72-0.85) | <0.001* |  |
|  | Underweight (n = 1,676) | 30/463 (6.5) | 137/1,213 (11.3) | 0.64 (0.40-1.00) | 0.051 | 0.085 |
|  | Normal weight (n = 21,216) | 375/4,136 (9.1) | 2,230/17,080 (13.1) | 0.71 (0.63-0.80) | <0.001* |  |
|  | Overweight (n = 17,097) | 317/3,177 (10.0) | 1,923/13,920 (13.8) | 0.75 (0.65-0.85) | <0.001* |  |
|  | Obese (n = 24,820) | 513/4,931 (10.4) | 2,704/19,889 (13.6) | 0.77 (0.69-0.86) | <0.001* |  |
|  | Non-smoker (n = 47,904) | 921/9,178 (10.0) | 5,208/38,726 (13.5) | 0.75 (0.69-0.81) | <0.001* | 0.214 |
|  | Past and current smoker (n = 16,905) | 314/3,529 (8.9) | 1,786/13,376 (13.4) | 0.72 (0.62-0.82) | <0.001* |  |
|  | Alcohol consumption > 1 time a week (n = 48,507) | 956/9,848 (9.7) | 5,084/38,659 (13.2) | 0.75 (0.69-0.81) | <0.001* | 0.530 |
|  | Alcohol consumption ≤ 1 time a week (n = 16,302) | 279/2,859 (9.8) | 1,910/13,443 (14.2) | 0.73 (0.63-0.84) | <0.001* |  |
|  | Non-COPD (n = 48,643) | 747/7,240 (10.3) | 5,591/41,403 (13.5) | 0.76 (0.70-0.83) | <0.001* | 0.067 |
|  | COPD (n = 16,166) | 488/5,467 (8.9) | 1,403/10,699 (13.1) | 0.70 (0.63-0.79) | <0.001* |  |
|  | Non-GERD (n = 45,951) | 321/9,342 (3.4) | 1,696/36,609 (4.6) | 0.77 (0.68-0.88) | <0.001* | 0.073 |
|  | GERD (n = 18,858) | 914/3,365 (27.2) | 5,298/15,493 (34.2) | 0.75 (0.69-0.82) | <0.001* |  |

Abbreviations: CI, confidence interval; CCI, Charlson comorbidity index; COPD, chronic obstructive pulmonary disease; DBP, diastolic blood pressure; GERD, gastro-esophageal reflux disease; NSAID, non-steroidal anti-inflammatory drug; OR, odds ratio; PPI, proton pump inhibitor; SBP, systolic blood pressure

* Un-conditional logistic regression model, Significance at P < 0.05

† Model 7 was adjusted for age, sex, income, region, total cholesterol, SBP, DBP, fasting blood glucose, obesity, smoking, alcohol consumption, CCI score, NSAID dates, H2 blocker dates, and COPD history, and number of GERD treated
